# Application of the Multi-Facet Rasch Model to Evaluate the Chief Resident Selection Survey: A Two-Year Study

**DOI:** 10.7759/cureus.16374

**Published:** 2021-07-13

**Authors:** Kadriye O Lewis, Haiqin Chen, Ross E Newman

**Affiliations:** 1 Department of Pediatrics, University of Missouri Kansas City School of Medicine, Children’s Mercy Hospital, Kansas City, USA; 2 Department of Testing Services, American Dental Association, Chicago, USA

**Keywords:** chief resident selection, chief resident leadership qualities, multi-facet rasch model, survey development and evaluation, raters’ severity or leniency

## Abstract

Introduction

The Chief Resident (CR) selection process is described by many residency programs as a collective effort from the residency program leadership, key faculty members, and resident peers. Unfortunately, the literature does not show any established guidelines, methods, or psychometric sound instruments to aid this process. The purpose of this study was to evaluate the properties of the newly developed CRs selection survey across two years using the Multi-Facet Rasch Model (MFRM).

Methods

This study used the MFRM to analyze two-year data from the newly developed CRs selection survey. After the first implementation of the tool in 2015, this instrument had its second-round evaluation process for the CRs selection in 2016. We applied a three-facet Rasch model (candidates, questions, and raters). We used Facets v. 3.66 and SAS 9.4 (SAS Institute Inc., Cary, NC) for data analysis.

Results

In 2015, 40 out of100 residents completed the survey to select three of the four candidates for the 2017-2018 CRs positions. The mean rating for each candidate showed that Candidate 1 received the highest rating of 5.56 while Candidates 2 and 4 received the exact same ratings. The majority of survey items performed very well based on the results from the MFRM while leaving room for improvement for a few items. In 2016, 55 out of 100 residents completed the revised survey to select three of the six candidates for the 2018-2019 CR positions. The mean rating showed that Candidate 3 received the highest mean rating of 5.81 while Candidate 2 received the lowest mean rating of 5.12. The item reliability was improved from 0.70 to 0.88 based on the results from the revised survey. The results were used to help inform decisions regarding the selection of chief residents.

Conclusions

The CR selection process requires a fair and collective effort from program leadership, relevant faculty members, and input from the resident group. Our study demonstrated that the survey tool we developed is appropriate to select CR candidates and MFRM is a promising technique in survey development and the evaluation of survey items.

## Introduction

Chief residents (CRs) are selected for a leadership position that offers them experience in the clinical, administrative, and educational activities of a hospital department or a residency program. Their leadership plays a critical role in post-graduate medical training in the context of exercising their administrative duties, teaching activities, and patient care [1]. In 1889, William Stewart Halsted, the father of the modern era of surgery, was the pioneer in establishing the concept of the chief residency in academic residency training programs when he was the Chairman of Surgery at the Johns Hopkins Hospital [2-3]. Since then, almost all academic centers designate or appoint a few of their high achieving resident physicians to spend either their final year or an additional year as CRs. The duties for CRs may vary depending on the size of the department, the length of the residency, and the number of housestaff members.

Although being selected as a CR is an honor and privilege sought by many residents at their respective programs, the literature does not identify any established guidelines, methods, or psychometrically sound instruments to augment the selection process. We only noted a few studies in the limited literature. One study conducted by Jain et al. surveyed 83 program directors regarding selection methods, duties, training, and evaluation of their CRs [4]. In this study, the authors showed that CRs were mostly appointed by their program directors or chairman, or through the vote of the faculty. Another study by Young et al. reported that the most common methods of CR selection were faculty vote (45%), combined resident-faculty vote (22%), and other (33%) [3]. On the other hand, many residency program websites describe the CR selection process as a collective effort between the Residency Program Director and key faculty members while also taking into account the input from resident peers; however, a formal outline of the process is not shared and specific tools, such as a validated survey to appropriate capture peer feedback, are not described. In essence, CRs selection typically lacks a reliable process to capture the performance and leadership qualities of potential candidates.

In our Pediatric Residency Program, we annually select three CRs during their postgraduate year (PGY)-2 year for PGY-4. Candidates self-nominate themselves submitting their biosketches that include a) background information, b) interests, c) professional plans beyond residency, d) goal statement, e) qualities the candidates possess that would make them a good CR, and f) goals for the CR position. Each candidate goes through two sets of interviews with the residency program leadership: 1) 30-minute themed interviews with Residency Program Director, Chair of Graduate Medical Education (GME), and Vice-Chair of GME; and 2) 30-minute group interviews with Associate Program Directors, Current Chief Residents, and Program Coordinators, and finally a resident survey to capture peer opinions on the CR candidates’ skills. While this model facilitates annual deliberation for the CR selection, the previous ranking survey reflected the popularity of the CR candidates among peers and was not able to capture personal and leadership characteristics desired in CR candidates. Therefore, we developed a unique survey instrument to capture those characteristics from the resident peers’ perspectives. We intended to use the results of this survey during the CR selection consensus meeting that is scheduled after all interviews are completed. Thus, the purpose of this study was to investigate the properties of the newly developed CRs selection survey tool under the item response theory (IRT) framework [5-6], specifically, we utilized a multi-facet Rash model (MFRM), which is an extended version of one-parameter IRT technique [7-8], focusing on three aspects of our survey tool: 1) Reliability of the items included in the first version and the revised version of the survey to differentiate each candidate for the CR position across two years of study, 2) performance of each item in respect of rating scales function in both version of the survey, and 3) raters’ severity or leniency in rating each candidate.

## Materials and methods

This survey-based study was conducted at the Pediatric Residency Program at Children’s Mercy Hospital in Kansas City, Missouri. Using the Multi-Facet Rasch Model (MFRM), we evaluated our CR selection survey tool from the resident peers’ perspectives, as an addition to the traditional CR selection interview process. After the utilization of the results in the first implementation for the CR selection in 2015, this instrument had its second-round development process for the CRs selection in 2016.

We obtained the Children’s Mercy Hospital Institutional Review Board approval 15100469 as non-human subjects for this study.

Tool development

To develop the CR selection survey, we conducted a comprehensive search within the Google, MEDLINE, PubMed Central, Scopus, PsycINFO, and ERIC databases to locate a validated tool that can measure the personal and leadership characteristics of a CR candidate. While we were not able to find well-established guidelines, methods, or psychometric instruments for selecting CRs in residency programs, this literature search enabled us to prepare a comprehensive list of desirable personal and leadership attributes of a person for a leadership position. Feedback from residency leadership then helped us determine key constructs with specific domains for measurement items for the CR tool. After the development of the first version of the survey instrument in 2015, we edited and finalized the items based on the feedback obtained from stakeholders.

The CR selection survey tool had two parts. The first consisted of two questions, rating 16 descriptive characteristics with a six-point quality scale from one representing “Very Poor” to six representing “Excellent” followed by an open-ended question. We used a six-point scale to force more positive or more negative response choices from respondents. All the items are listed alphabetically so as not to give any priority to any of the traits and qualities. The second part of the survey has two rating-type questions with a comment box provided. After the implementation of this survey, we revised it for the next cycle in 2016 based on the feedback from program leadership involved in the CRs selection process. In this version, while we revised a few items in the first part of the survey, we also added new items to further measure candidates’ characteristics and leadership skills that increased the number of the items from 16 to 22 (Appendix 1). We obtained the face and content validity of the tool through peer reviews in both versions.

Data collection

Using SurveyMonkey as our online data collection platform, we implemented the first version of the survey in September 2015 to identify candidates for the 2017-2018 CR positions. We used the revised version of the survey in September 2016 for the selection of 2018-2019 CRs. We emailed the survey link for anonymous responses to all pediatric residents in our program with two follow-up reminders for both implementations. Along with the survey link, we also provided the candidates’ biosketches/CVs in our internal shared folder to allow the respondents further information about each candidate. There were four chief resident candidates in 2015 and six candidates in 2016. In our institution, all candidates are self-nominated for the CR positions and their peers served as the raters in this study. Survey results are only reviewed by program leadership involved in CR selection and not available to CR candidates or peers.

Data analysis

Traditionally, when analyzing survey questionnaires, a sum score or average score over all the survey questions are often used. However, respondents, also called raters if using a rating scale in the survey, may have a different threshold or may use different criteria when they respond to survey questions. For example, it may have a different meaning when respondent 1 and respondent 2 both selected “strongly agree” to the same survey question. Similarly, some respondents may indorse “Poor” after comparing all candidates (i.e., relative ranking) while others may just indorse “Poor” but not thinking of any other candidates (i.e., absolute ranking). This is called the rater effect or more accurately rater leniency or stringency in the literature. This rater effect cannot be captured if using traditional average/sum scores.

In this study, we utilized the multi-facet Rasch model (MFRM) to address how the rater effect can be captured when analyzing the survey data. Thus, we applied a three-facet Rasch model to our study, including 1) Candidates; 2) Questions; and 3) Raters. MFRM is intended to calibrate the candidate ability or performance (corresponding to the sum or average score over all the survey items for each candidate), item difficulty (corresponding to the sum or average score over all the candidates and raters for each item), and rater stringency or leniency (corresponding to the sum or average score over all the candidates and items for each rater) into the same logit scale, facilitating direct comparison between elements. In the analyses, the item facet was centered on zero in the logit scale so that the candidate performance and rater stringency or leniency could be evaluated relative to the items. The advantage of MFRM is that all sources of variability, such as candidates, survey questions, and raters, which all have the potential to influence measurement results, are considered simultaneously [9].

We first analyzed the 2015 survey results to evaluate the ability of CRs and to assess the quality of survey items. Then we analyzed the 2016 survey results using a similar method as in 2015 to evaluate the candidate’s ability and to assess the quality of the revised survey items. For each year data, we provided the results of:

1) The chi-square test: It evaluates the divergence between observed and model expected scores [10]. We reported these test statistics as a regular check of the overall data model fit. However, it would not be a primary concern whether it is statistically significant or not under the MFRM framework [11].

2) Separation reliability: The separation reliability is equivalent to Cronbach’s alpha, ranging from 0 to 1. According to Linacre [11], separation reliability for the source of rater variance is used in calculating the inter-rater agreement index. The separation reliability is actually a measure of how different the measures are within each facet examined in this study.

3) Measurement results of each element and each facet: In addition to the observed average ratings as in other studies, the measurement in the logit scale and its associated standard error are also provided from the MFRM outputs. These are direct measures of performance of each candidate, survey question, and rater.

4) Infit and outfit statistics: Analysis of infit and outfit statistics are two fit indices that represent the relationship between observed ratings and model-derived ratings for both items and candidates. Statistics equal to or near 1 show perfect correspondence between observed and expected values while standardized values higher than 2 signal the presence of serious distortions in the data [12-13]. The difference between the infit and outfit values derives from the way in which the statistics are calculated. The outfit is sensitive to the outlying or extreme ratings.

All these statistics mentioned above were reported for candidates, items, and raters, respectively. In addition, the interaction analyses between candidates and items were also assessed to provide additional information for candidates’ performance and survey items. Data analysis of MFRM was completed using Facets v. 3.66.1 [14]. The remaining data analysis was completed using SAS 9.4 (SAS Institute Inc., Cary, NC).

## Results

Descriptive results

Out of 100 residents, 40 of them responded to the 2015 survey to select three of the four candidates for the position of CRs for the year 2017-2018. Table 1 shows the frequencies of 16 survey questions at each rating, with mean ratings and their associated standard deviation for each candidate. The mean rating for each candidate showed that Candidate 1 received the highest rating of 5.56 while Candidates 2 and 4 received the exact same meaning ratings over 16 survey questions.

**Table 1:** Frequencies at each rating and mean ratings across 16 survey questions in 2015 administration

Candidate	Very Poor (1)	Poor (2)	Below Average (3)	Average (4)	Very Good (5)	Excellent (6)	Mean Rating (SD)
Candidate 1	0	0	0	28	174	320	5.56 (0.60)
Candidate 2	0	2	16	89	167	273	5.27 (0.86)
Candidate 3	0	0	1	40	171	196	5.38 (0.67)
Candidate 4	0	0	5	57	188	184	5.27 (0.73)

In 2016, a total of 55 raters out of 100 residents completed the revised CRs’ selection survey to select three of the six candidates for the position of CRs for the year 2018-2019. Table 2 displays the frequencies of 22 survey questions at each rating, with mean and standard deviation for each candidate. The mean rating for the 2016 survey results showed that Candidate 3 received the highest mean rating of 5.81 while Candidate 2 received the lowest mean rating of 5.12.

**Table 2:** Frequencies at each rating and mean ratings across 22 survey questions in 2016 administration

Candidate	Very Poor (1)	Poor (2)	Below Average (3)	Average (4)	Very Good (5)	Excellent (6)	Mean Rating (SD)
Candidate 1	0	0	8	59	283	663	5.58 (0.64)
Candidate 2	0	0	19	160	349	301	5.12 (0.80)
Candidate 3	0	0	0	2	131	564	5.81 (0.40)
Candidate 4	0	4	21	129	344	412	5.25 (0.81)
Candidate 5	0	0	2	24	180	530	5.68 (0.55)
Candidate 6	0	2	3	43	348	357	5.40 (0.64)

2015 survey results from the MFRM

For the person and model fit, the chi-square test is 𝑥2 (3) = 76.2, p < 0.001, with a candidate reliability separation index of 0.96. A reliability separation index of 0.70 would suggest that, on average, there are discernible statistically significant differences between candidates. Therefore, both the chi-square test and the reliability separation index indicate that overall, the model fits the data well and candidates’ abilities could also be differentiated well. Table 3 provides summary statistics for the candidate measure report from FACETs. Candidate 1 and Candidate 3 received relatively high performance with a measure of 3.61 and 3.15 on a logit scale while Candidate 2 and Candidate 4 received about the same measure with 2.74 and 2.73, respectively (Table 3). The infit and outfit results for each candidate are not of the primary concern in this study since there are only four candidates.

**Table 3:** Candidate measure report from FACETs

Candidate	Observed Average	Measure (SE)	Infit		Outfit	
			MnSq	ZStd	MnSq	Zstd
Candidate 1	5.56	3.61 (.09)	.99	-.1	1.00	0
Candidate 2	5.38	2.74 (.07)	1.24	3.6	1.24	2.7
Candidate 3	5.27	3.15 (.09)	.83	-2.5	.94	-.5
Candidate 4	5.27	2.73 (.08)	.87	-2.0	.82	-2.2

For the item and model fit, the chi-square test is 𝑥2 (15) = 49.6, p < 0.001, and the item reliability is 0.70. We expect some degree of differences among items so that each item could contribute to the overall survey instrument. Table 4 provides the observed average, logit item difficulty with standard error, and item infit and outfit statistics. Item difficulty measure ranges from -0.44 (easiest item) for item 3 (“respectful”) to 0.51 (most difficult) for item 8 (“acceptance of criticism/ability to accept his/her own mistakes”). If by using the criteria of the standardized value of 2 (Zstd) indicating a poor fit, then the infit and/or outfit column in Table 4 shows that the item 4 (“confident”), item 10 (“diversity awareness/open-minded”), and item 11 (“enthusiastic”) exhibit a relatively poor item fit, indicating these three items do not fit the model well.

**Table 4:** Item measure report from FACETs for the 2015 survey data

Question	Observed Average	Measure (SE)	Infit		Outfit	
			MnSq	ZStd	MnSq	Zstd
1	5.35	-0.02 (.16)	0.9	-0.7	0.87	-0.6
2	5.25	0.22 (.16)	1.01	0	1.34	1.8
3	5.49	-0.44 (.17)	0.92	-0.5	0.91	-0.3
4	5.25	0.29 (.15)	1.35	2.4	1.6	2.9
5	5.39	-0.11 (.16)	0.91	-0.6	0.8	-1
6	5.42	-0.19 (.16)	0.91	-0.6	0.95	-0.1
7	5.36	-0.07 (.16)	0.8	-1.5	0.76	-1.3
8	5.13	0.51 (.16)	1.1	0.7	1.17	1
9	5.26	0.2 (.16)	1.21	1.5	1.15	0.9
10	5.39	-0.19 (.17)	0.68	-2.5	0.62	-2.2
11	5.23	0.3 (.16)	1.32	2.2	1.34	1.8
12	5.20	0.36 (.16)	0.94	-0.4	0.89	-0.5
13	5.48	-0.4 (.17)	1.1	0.7	1.25	1.1
14	5.29	0.17 (.16)	0.92	-0.5	0.86	-0.7
15	5.42	-0.25 (.18)	0.9	-0.6	0.83	-0.8
16	5.46	-0.38 (.17)	0.9	-0.7	0.85	-0.6

For the overall rater and model fit, the chi-square test is 𝑥2 (39) = 729.6, p < 0.001, signifying that the raters did not all exercise the same level of severity when evaluating these four candidates in this study. The reliability of the rater separation index is 0.89 if extreme ratings were included. This result suggests that there are discernible statistically significant differences between at least two of the raters. At the very least, the most severe and most lenient raters were significantly different. Therefore, both the chi-square best and reliability of the rater separation index suggest the need for the adjustment of severity when assessing candidates’ performance. Rater severity levels range from -4 (most lenient) to 3.41 (most severe) on the logit scale.

**Figure 1:**
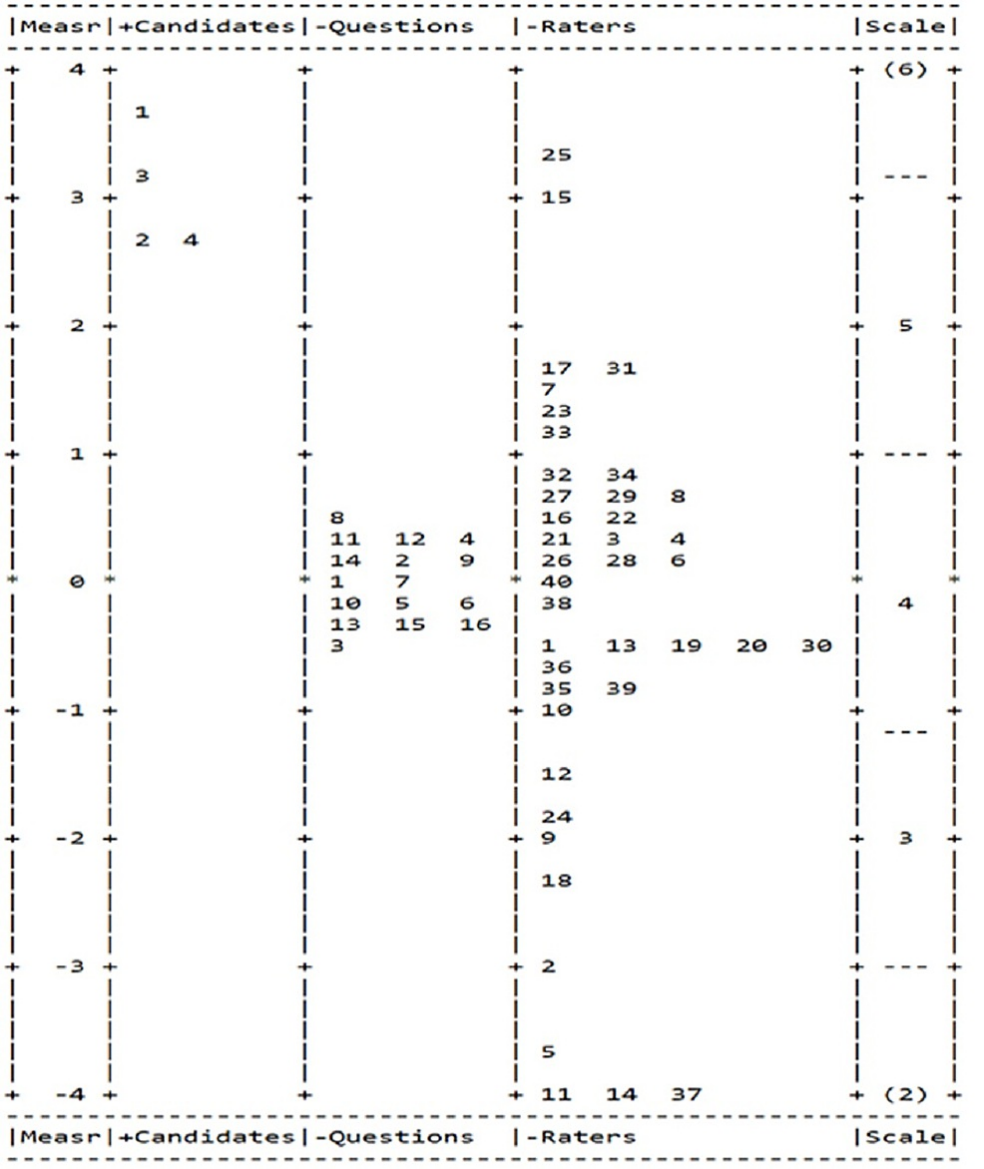
Candidates, questions, and raters map from the three-FACET Rasch analysis for the 2015 survey

In addition, the FACETS variable map (also called “Wright map”) is particularly useful (Figure 1) when interpreting the MFRM results. This map visually summarizes all facets investigated, with each facet presented in a separate column. The logit scale appears in the first column on the map. The other columns are candidates (the second column), questions (the third column), raters (the fourth column). Only the first facet (candidates) is positively oriented (+ Candidates), which means more able candidates have positive measurement values and are therefore placed up in the column while less able ones are placed lower in the column. For the questions column, more difficult questions are placed on the top while easy questions are on the bottom. For the raters’ column, more severe raters appear higher in the column while more lenient raters appear lower. The variable map allows for different comparisons within and between facets. As can be seen, the variability across raters in their level of severity was substantial. In fact, the raters’ effect is very spread, with a value ranging from around -4 (most lenient for raters of 11, 14, and 37) to around 3.5 (most severity for raters of 15 and 25).

**Figure 2:**
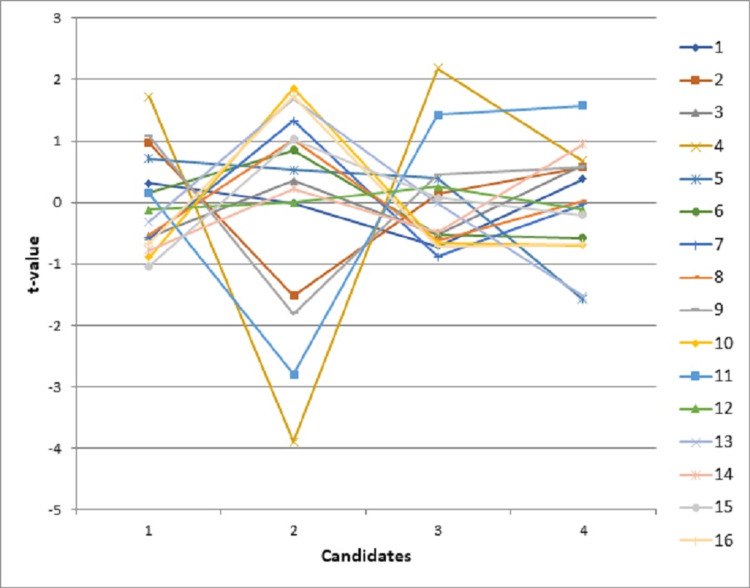
Relative measures based on the candidates and questions interaction analysis for the 2015 survey

The interaction between candidates and survey questions is particularly useful to provide additional information that the observed rating candidates received on a particular item were unexpected from the model. The relative measures (t scores) above 2.0 or below -2.0 would indicate an interaction effect. Figure 2 shows the relative measures for the candidates and questions interaction. Candidate 1 and Candidate 3 received relatively consistent ratings across all 16 questions. However, Candidate 2 received particularly low ratings on question 4 (confident), question 9 (common sense), and question 11 (efficient). On closer examination of the interaction between raters and candidates, five raters or more endorsed particularly low ratings to Candidate 2 (the results table was not provided since there are 40 raters). This piece of information might be particularly useful when the program had to select a CR candidate between Candidate 2 and Candidate 4.

In summary, the results showed that this newly developed survey can differentiate CR candidates’ performance well. Based on the ability estimates from MFRM, Candidate 1 and Candidate 3 were rated higher than Candidate 2 and Candidate 4 by their peer residents. As for Candidate 2 and Candidate 4, although they received about the same ratings either based on the mean ratings or based on the ability estimates from MFRM, Candidate 4 would be a better fit than Candidate 2 if an additional candidate had to be selected. In terms of item performance for this newly developed CR selection tool, the majority of items performed very well based on the psychometric results from the MFRM while leaving room for improvement for a few items.

2016 survey results from the MFRM

For person and model fit, the chi-square test is 𝑥2 (5) = 753.5, p < 0.001, with a candidate reliability of 0.99. Similar to the survey results in 2015, both the chi-square test and the reliability separation index suggest that overall, the model fits the data, and candidates’ abilities could be differentiated very well. The candidate measure report from MFRM (Table 5) showed that Candidate 3 received the highest ratings with an ability estimate of 4.33 on a logit scale followed by Candidate 5 and Candidate 1 with a logit scale of 3.48 and 3.05, respectively. Candidate 2 and Candidate 4 received relatively lower ratings among all six candidates with ability estimates of 1.96 and 2.05, respectively.

**Table 5:** Candidate measure report from FACETs for the 2016 survey

Candidate	Observed Average	Measure (SE)	Infit		Outfit	
			MnSq	ZStd	MnSq	Zstd
Candidate 1	5.58	3.05 (.06)	1.01	.2	.96	-.6
Candidate 2	5.12	1.96 (.05)	.91	-1.8	.89	-2.2
Candidate 3	5.81	4.33 (.10)	.89	-1.3	1.10	.7
Candidate 4	5.25	2.05 (.05)	1.29	5.1	1.28	5.0
Candidate 5	5.68	3.48 (.08)	.91	-1.4	.84	-1.8
Candidate 6	5.40	2.58 (.06)	.94	-.9	.98	-.3

For the item and model fit, the chi-square test is p < 0.001, with a reliability of .88. Table 6 provides the observed average logit item difficulty with standard error and item infit and outfit statistics. The logit measure of item difficulty ranges from -0.76 (easiest item) for item 8 (dedicated) to 0.62 (hardest item) for item 2 (ability to accept his/her own mistakes). In addition, the examination of the infit and outfit statistics showed that item 3 (assertive), item 6 (confident), and item 17 (non-discriminatory) showed relatively poor item fit with a mean square of 1.88, 1.7, and 0.68, indicating these three items do not fit the model well and do not contribute to assessing candidates’ performance.

**Table 6:** Item measure report from FACETs for the 2016 survey

Question	Observed Average	Measure (SE)	Infit		Outfit	
			MnSq	ZStd	MnSq	Zstd
1	5.38	0.16 (.11)	0.88	-1	0.81	-1.5
2	5.20	0.62 (.11)	1.07	0.6	1.05	0.4
3	5.35	0.21 (.11)	1.88	6.6	2.45	8.4
4	5.53	-0.28 (.13)	0.92	-0.6	0.99	0
5	5.50	-0.18 (.12)	1.13	1.1	1.04	0.2
6	5.39	0.12 (.12)	1.7	5.4	1.73	4.7
7	5.44	0.01 (.12)	0.82	-1.7	0.73	-2.1
8	5.64	-0.74 (.14)	0.95	-0.3	0.79	-1.1
9	5.27	0.49 (.11)	1.2	1.8	1.1	0.8
10	5.47	-0.05 (.12)	0.96	-0.3	1.19	1.3
11	5.55	-0.33 (.13)	1.06	0.5	0.9	-0.6
12	5.34	0.32 (.12)	0.81	-1.8	0.8	-1.6
13	5.55	-0.33 (.13)	0.95	-0.4	0.85	-0.9
14	5.61	-0.55 (.14)	0.89	-0.9	1.11	0.6
15	5.52	-0.23 (.13)	0.78	-2	0.73	-1.9
16	5.40	0.16 (.12)	0.77	-2.2	0.72	-2.2
17	5.48	-0.1 (.13)	0.68	-3.2	0.61	-3.0
18	5.33	0.32 (.11)	1	0	0.98	-0.1
19	5.51	-0.2 (.13)	0.85	-1.4	1.01	0.1
20	5.22	0.61 (.11)	0.94	-0.5	0.91	-0.7
21	5.48	-0.14 (.12)	1.02	0.2	0.88	-0.8
22	5.41	0.11(.12)	0.87	-1.1	0.75	-2.0

For the overall rater and model fit, the chi-square test is 𝑥2 (53) = 1281.0, p < 0.001, and the reliability is 0.94. This result also suggests the need for the adjustment of severity when assessing candidates’ performance. Rater severity levels range from -2.17 (most lenient) to 2.26 (most severe) on the logit scale. Among all 55 raters, five raters endorsed the rating of 6 (“excellent”) for all six candidates and the questions they rated. These 5 raters’ ratings were weighted to the minimum by FACETS so that the candidates’ ability estimates were not affected that much by these extreme ratings. In addition, the three strictest raters endorsed the average observed average ratings of 4.4, 4.7, and 4.8 across all 22 questions and six candidates.

**Figure 3:**
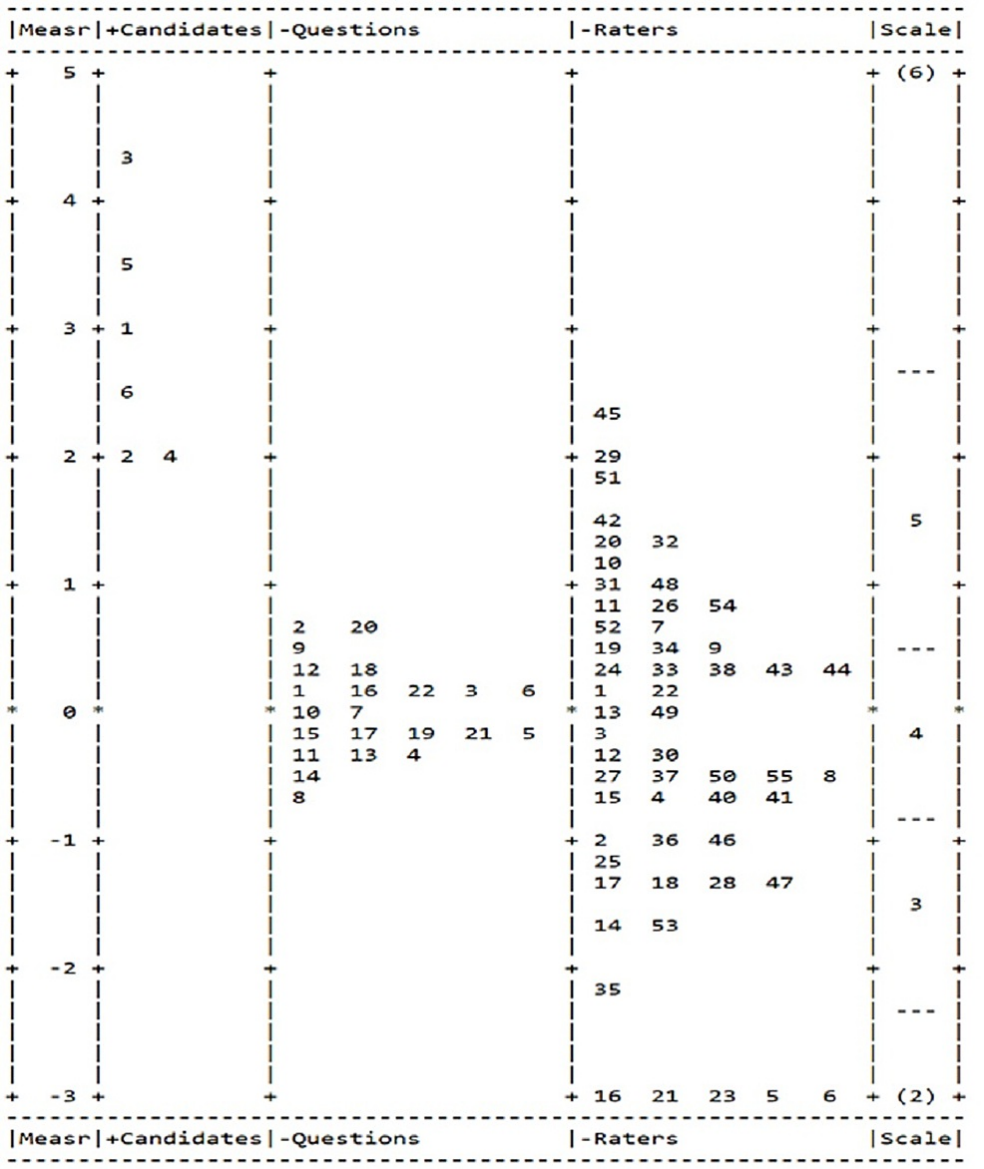
Candidates, question, raters map from the three-facet Rasch analysis for the 2016 survey.

We also provided results of the FACETS variable map so that the relative distribution of each facet can be seen (Figure 3) for the 2016 survey. The rater column still shows a greater departure of severity and leniency of raters on a logit. Five raters (5, 6, 16, 21, and 23) with similar but notably different positions at the bottom, indicating they are the most lenient raters with a logit scale of around -3. From this variable map, we also can see that raters 25, 49, and 51 were the most stringent raters and were placed on the top of the rater column. It is also similar to the results in 2015; -22 items were - placed between -1 to 1, indicating the items were not spread enough when compared to the variability of raters.

**Figure 4:**
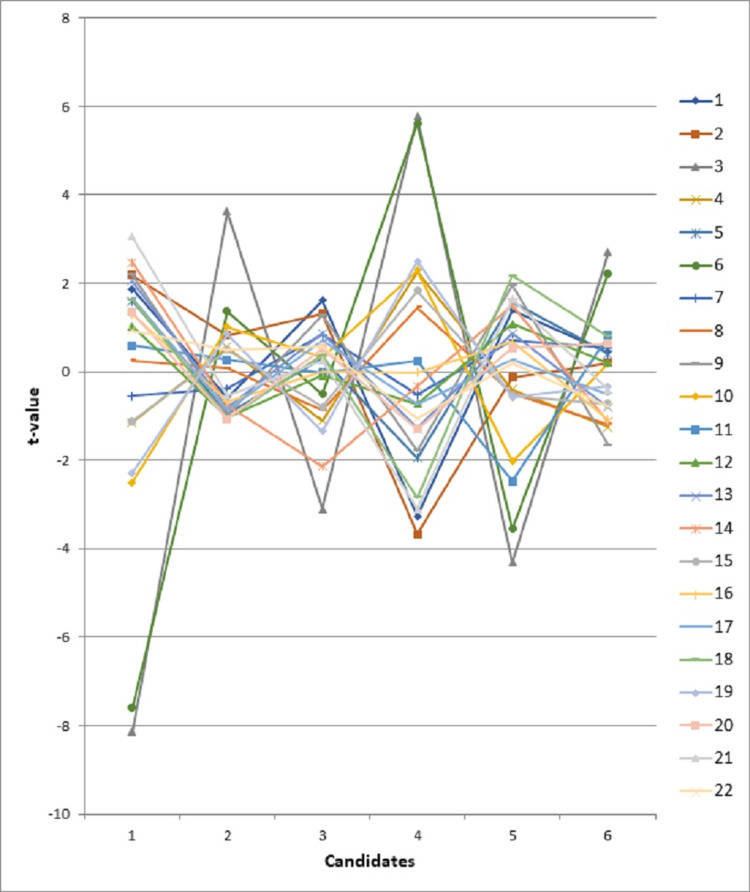
Candidates and questions interaction analysis for the 2016 survey

We also provided additional analysis of the interaction between candidates and survey questions (Figure 4). If using the criterion of the relative measures (t scores) above 2.0 or below -2.0, the results showed that Candidate 1 received low scores on question 3 (Assertive) and question 6 (Confident). On closer examination of the ratings of Candidate 1, we found that rater 20 provided extremely low scores on questions 3 and 6. In addition, Candidate 5 also received a very low score on item 4 (Attentive) and item 19 (Problem-solving), which were endorsed by rater 1 and rater 52.

In summary, the results from the revised survey showed that the item reliability was improved from 0.70 to 0.88. This revised survey can differentiate CR candidates’ performance well. Based on the ability estimates from MFRM, Candidate 3, Candidate 5, and Candidate 1 would be recommended for chief residents.

## Discussion

Our study demonstrated the development process of a psychometrically sound survey for CR selection through the utility of MFRM. When combined with our full CR selection process, including utilizing biosketches to review candidate self-identified skills and goals for the position; interview strength with all program leadership; and the MFRM showing peer identified leadership qualities, the bundle of all elements allowed for comprehensive CR selection. The overall candidate ranking results from MFRM are almost identical to the ranking results if using the average score, suggesting that MFRM results are valid and consistent with the traditional method. However, it would be hard to differentiate Candidates 3 and 4 for the 2015 survey if using traditional average score/sum score to evaluate each candidate. A major benefit of applying the MFRM to analyze the survey data is that the rater’s severity/leniency can be treated as one facet as survey items and CR candidates, and then unbiased ratings can be applied to the item and candidate performance evaluation. Another benefit of using MFRM is that it can provide multiple measures to assess the quality of each item, which is very useful in survey development [11]. Therefore, MFRM makes important contributions to the analysis of survey results by simultaneously assessing CR candidates, the survey items, and rater severity and leniency effect.

The findings are promising since the results showed that the survey in both years is reliable and can differentiate candidates’ personal and leadership attributes well. Meanwhile, the innovative application of MFRM provides us insights into the survey development by providing a measure and related infit and outfit statistics for each item. If one with extreme infit/outfit statistics, this item could be edited for clarity or be removed from the survey. The MRFM provides a common metric for the facet scores, including candidates’ performance, survey questions, and raters so that item quality and raters’ severity/leniency can be monitored. This innovative way could facilitate our understanding of the scale development process as well as provide objective measurement of each facet evaluated in this model.

Reliability of the survey items

Our CR selection survey tool and MRFM model allowed the CR selection process to capture the personal and leadership characteristics of CR candidates from the resident peers’ perspectives. Three facets of the modeling enabled us to revise the items after the first implementation of the CR selection in 2015. The second round of the CR selection in 2016 provided additional evidence to go over another iteration of the items for further improvement of the tool. The chi-square tests and separation reliability index were used to measure the overall data-model fit results for candidates, survey questions, and raters for the surveys in 2015 and 2016. The results obtained through MFRM demonstrated that the survey overall can differentiate candidates well in both years and the majority of items performed very well in differentiating candidates within each year. Although, in general, a low separation of rater reliability is more desirable, indicating less variability across raters. A high value of separation reliability would indicate the existence of rater severity and leniency effects by the MFRM [15].

Performance of rating scales

The infit and outfit statistics indicate the goodness of fit of items to the latent trait or candidate performance and allow us to select the more reliable items. Misfit items reflect on large infit or outfit values [16]. The infit or outfit values, which greatly deviate from the value of 1, would indicate that some unexpected ratings occurred for this item: either a low rating was indorsed to a relatively easy item (with negative logit) or a high rating was endorsed to a relatively difficult item (with high positive logit). Based on misfit rating information provided by FACETS for the survey in 2015, the relative misfit items in 2015 were either removed or edited; the survey went over another iteration in 2016. In general, the overall item fit was improved for the 2016 survey except for a few relative misfit items of the survey in 2016. This leaves room for further improvement of this CR survey. However, the variability of survey items for the survey of 2015 and 2016 is relatively small in terms of item easiness and item difficulty, which leaves room for improvement in future survey development.

Raters’ severity or leniency

Greater emphasis should be placed on the differences in the severity of each respondent/rater may have. This study goes beyond the mean or sum scores over all the survey items by applying the MFRM model. The MFRM puts the candidates, survey questions, raters’ severity, or leniency into the same scale so that the raters’ facet becomes independent from survey questions and the candidate’s performance. Then the rater severity/leniency could be taken into consideration by the statistical model [11]. The pronounced variation in rater fit statistics, including the chi-square test and separation reliability index, suggested that, in general, the raters varied when they rated the candidates. In addition, this study showed the rater effect ranging from around -4 to around 4 for the survey in 2015 (Figure 1) and around -3 to around 2.5 for the survey in 2016 (Figure 3). This result confirmed our concern that the rater’s severity and leniency should be addressed when using the rating scale in the survey development process. Many factors may contribute to a rater’s tendency to rate harshly or leniently. For example, the severity and leniency tendency may be due to the raters’ professional experience, personality traits, attitudes, or familiarity with CR candidates.

Overall, our efforts with a new approach to gathering validity evidence for a chief resident selection survey have added value and produced significant useful data for the CR selection team. The overarching aim was to improve the process to identify the best candidates with personal and leadership characteristics from the resident peers’ perspectives.

Limitations of the study

Our study has some limitations that need to be pointed out. This study only collected data from one institution. However, the chief resident selection study, by nature, can hardly have tens or hundreds of candidates within one institution. It also would not make sense to select, compare, and evaluate chief resident candidates across several institutions. The data matrix in this study we analyzed is 40 (residents) * 4 (candidates) * 16 (questions) in 2015, and 55 (residents) * 22 (questions) * 6 candidates in 2016, which is not small when compared to other survey studies in the field. In addition, a relatively large number of item change (from 16 questions to 22 questions) should be avoided in future practice. The ideal survey development process is to revise those poor fit items identified by MFRM and administer the survey again so that we can monitor the performance of the survey items and keep improving the survey during each administration. We also noticed that the “very poor” rating scale category was never selected for all questions by any of the raters in both years while the “poor” category only was selected by very few raters. This leaves room for discussion whether the “Very poor’ or “Poor” rating categories should be included in the survey or not. In the survey development, we would expect the symmetric design of the rating category. However, for this specific chief resident selection purpose, most candidates were nominated because of their excellent performance and standing in the program. We also can consider including the “Very Poor” and “Poor” rating categories because both the number of candidates and raters are relatively small for both years of administrating the surveys. With a relatively larger sample size of candidates or raters, we may see more endorsement of “poor” or “very poor” if this survey is administrated to multiple institutions in the future. In addition, we do not know the exact process of how peer residents would evaluate these CR candidates. If they evaluate all CR candidates’ relative performance, then it is possible that they may endorse “Very Poor” or “Poor” to specific CR candidates on certain survey items. Besides removing these two rating categories, the other way to remediate this if each rating category can be clearly defined and differentiated in the survey.

## Conclusions

Looking for an effective and fair way to select CRs and to identify personal and leadership attributes are our primary objectives. In this regard, our surveys for collecting candid feedback from peer residents were instrumental to identify the desired characteristics in residents. This process was important since the information about the candidate’s personal, professional, and leadership qualities gave us insights into each candidate’s abilities to take a CR leadership role. Our study demonstrated an application of multi-facet Rasch modeling to analyze survey data and to inform decisions regarding the selection of CRs.

Finally, the newly designed CR selection survey development process we adopted in this study can be applicable to other educational settings and/or professionals, especially when the selection process requires a collective effort from multiple parties (faculty, peers, trainees, program directors, or other stakeholders). In addition, this model can increase the transparency in the selection processes by adjusting potential rater severity and leniency. In the meanwhile, this paper also motivates researchers in the field to apply MFRM to validate the survey and to analyze the Likert scale survey data.

## Appendices

Chief residents selection survey

Chief residents serve as key leaders with several roles and provide a variety of functions in our Pediatric Residency Program at Children’s Mercy Hospital. Now it is time for us to select the Pediatric Chief Residents for the [date] academic year.

We will select three chiefs based on their clinical excellence, humanity, professionalism, teaching and leadership skills. The ideal chief resident should be well organized, attentive to detail, approachable, skilled at teaching, and extremely knowledgeable of pediatric medicine. S/he should also possess excellent communication skills for conflict management, clarity of thought, and confidence, interacting with applicants, residents, faculty, and administration, representing our program well at all times.

For the [xxx academic year], we have the following six candidates for the Chief Resident positions:

1.      (Name of the Candidate)

2.      (Name of the Candidate)

3.      (Name of the Candidate)

4.      (Name of the Candidate)

5.      (Name of the Candidate)

6.      (Name of the Candidate)

The candidates’ biosketches can be found in the “Housestaff Shared Drive” under the “Chief Bios” folder.

Participation in this survey is anonymous, which means that no identifying information about you will be collected, so getting candid feedback from you is very important for us to identify the right persons for the Chief Resident positions. Please take a few minutes to evaluate each candidate and respond to this survey by [date].

If you have any questions regarding this survey, please email [contact name and email address].

Thank you, in advance, for your participation.

(Name of the Candidate)

1.  Please help us understand how you perceive this candidate. Given the following characteristics, please rate them from 1 to 6.

Scale: Very Poor (1), Poor (2), Below Average (3), Average (4), Very Good (5), Excellent (6)

Ability to work with others/ relationship-building

Ability to accept his/her own mistakes

Assertive

Attentive

Attitude towards residents

Confident

Collaborative

Dedicated

Diplomatic/Tactful

Efficient/Resourceful

Enthusiastic

Fair-minded/unbiased

Friendliness/Kindness/Sense of Humor

Integrity and honesty

Judgment and Decision-making

Listening for ideas, not just words

Nondiscriminatory

Patience /tolerance

Problem-solving

Receptive of criticism

Respectful/Considerate/ Courteous

Self-awareness and Self-Discipline

What are some descriptive words that come to mind when you think of (Name of the Candidate)?

Do you have any reservations about (Name of the Candidate) becoming the Chief Resident?

Yes     No

If yes, please specify (Serious consideration will be given to your comments):_______________

2. Overall, which three do you feel are the most outstanding candidates for the Chief Resident positions (SELECT ONLY THREE)?

(Name of the Candidates)

3. How well do you know each candidate?

Scale: Very Well, Average, Acquainted, Not very Well

(Names of the Candidate)

Thank you for taking the time to complete this survey!

## References

[1] Dabrow SM, Harris EJ, Maldonado LA, Gereige RS (2011). Two perspectives on the educational and administrative roles of the pediatric chief resident. J Grad Med Educ.

[2] Grant I, Dorus W, McGlashan T, Perry S, Sherman R (1974). The chief resident in psychiatry. Arch Gen Psychiatry.

[3] Young MA, Stiens SA, Hsu P (1996). Chief residency in PM&R. A balance of education and administration. Am J Phys Med Rehabil.

[4] Jain SS, DeLisa JA, Campagnolo D (1993). Chief residents in physiatry. Expectations v training. Am J Phys Med Rehabil.

[5] Bock RD (1997). A brief history of item theory response. Educ Meas Issues Pract.

[6] de Ayala RJ (2009). The Theory and Practice of Item Response Theory. https://www.guilford.com/books/The-Theory-and-Practice-of-Item-Response-Theory/R-de-Ayala/9781593858698/reviews.

[7] Myford CM, Wolfe EW (2004). Detecting and measuring rater effects using many-facet Rasch measurement: part II. J Appl Meas.

[8] Bond TG, Fox CM Applying the Rasch Model: Fundamental Measurement in the Human Sciences, 2nd Ed. Sciences (2nd.

[9] Lunz ME, Stahl JA (2021). Severity of grading across time periods. ERIC.

[10] Rodel E (1970). Fisher RA: Statistical Methods for Research Workers, 14. Aufl., Oliver & Boyd, Edinburgh, London 1970. XIII, 362 S., 12 Abb., 74 Tab., 40 s. Biom Z.

[11] Eckes T (2009). Multi-facet Rasch measurement. Reference Supplement to the Manual for Relating Language Examinations to the Common European Framework of Reference for Languages: Learning, Teaching, Assessment (Section H).

[12] Linacre JM (2021). A User's Guide to FACETS Rasch-Model Computer Programs. Chicago: Winsteps.com.

[13] Wright BD, Linacre JM, Gustafson JE, Martin-Lof P (2021). Reasonable mean-square fit values. Rasch Measurement Transactions.

[14] Linacre JM (2009). Facets Rasch measurement computer program. https://www.winsteps.com/winsteps.htm.

[15] Han C (2021). Detecting and measuring rater effects in interpreting assessment: a methodological comparison of classical test theory, generalizability theory, and many-Facet Rasch measurement. Testing and Assessment of Interpreting.

[16] Wind S, Hua C (2021). Rasch Measurement Theory Analysis in R: Illustrations and Practical Guidance for Researchers and Practitioners. Illustrations and Practical Guidance for Researchers and Practitioners.

